# Comparison of construct validity of two short forms of Stroke-Specific Quality of Life scale

**DOI:** 10.1371/journal.pone.0188478

**Published:** 2017-12-06

**Authors:** Chia-Yeh Chou, Chien-Yu Huang, Yi-Jing Huang, Gong-Hong Lin, Sheau-Ling Huang, Shu-Chun Lee, Ching-Lin Hsieh

**Affiliations:** 1 Department of Occupational Therapy, College of Medicine, Fu-Jen Catholic University, New Taipei City, Taiwan; 2 School of Occupational Therapy, College of Medicine, National Taiwan University, Taipei City, Taiwan; 3 Department of Occupational Therapy, I-Shou University, Kaohsiung County, Taiwan; 4 Department of Physical Medicine and Rehabilitation, National Taiwan University Hospital, Taipei City, Taiwan; 5 Taipei City Psychiatric Center, Taipei City Hospital, Taipei, Taiwan; 6 Department of Occupational Therapy, College of Medical and Health Science, Asia University, Taichung City, Taiwan; Universiti Sains Malaysia, MALAYSIA

## Abstract

**Background:**

No studies have compared the 2-factor structures of Wong’s and Post’s versions of the short-form Stroke-Specific Quality of Life (i.e., 12-item SSQOL) scale. This study compared the construct validity of 2 short-forms of the 12-item-SSQOL (not the 12-domain-SSQOL).

**Methods:**

Data were obtained from a previous validation study of the original 49-item SSQOL in 263 patients. Construct validity was tested by confirmatory factor analysis (CFA) to examine whether the two-factor structure, including psychosocial and physical domains, was supported in both versions. The CFA tested the data-model fit by indices: chi-square χ^2^/df ratio, root mean square error of approximation (RMSEA), comparative fit index (CFI), nonnormative fit index (NNFI), standard root mean square residual (SRMR), and parsimony normed fit index (PNFI). Item factor loadings (cutoffs: .50) were examined. Model fit was compared using Akaike information criterion (AIC) and consistent AIC (i.e., CAIC) values.

**Results:**

All model fit indices for Post’s version fell within expected ranges: χ^2^/df ratio = 2.02, RMSEA = 0.05, CFI = 0.97, NNFI = 0.97, SRMR = 0.06, and PNFI = 0.76. In the psychosocial domain, the item factor loadings ranged from 0.46 to 0.63. In the physical domain, all items (except the language and vision items) had acceptable factor loadings (0.68 to 0.88). However, in Wong’s version, none of the model indices met the criteria for good fit. In model fit comparisons, Post’s version had smaller AIC and CAIC values than did Wong’s version.

**Conclusions:**

All fit indices supported Post’s version, but not Wong’s version. The construct validity of Post’s version with a 2-factor structure was confirmed, and this version of the 12-item SSQOL is recommended.

## Introduction

Health-related quality of life (HRQOL) is considered an important outcome measurement [1] and is intended to assess individual’s perceived functioning regarding the different effects of a disease and/or intervention [2]. The subjective perspectives captured in HRQOL usually include multiple domains, particularly the physical, psychological, and social domains [2, 3]. These domains determine whether there are deficits in physical, psychological, or social function, such as those frequently manifested in patients with stroke. Thus, measures of HRQOL are useful for assessing the HRQOL of patients with stroke.

Construct validity can be defined as whether a measure captures the hypothesized or underlying construct(s) it is intended to measure [4–6]. Good construct validity is required for a measure to provide valid assessments. The construct validity of a HRQOL measure can be empirically validated by examining its factor structure [7]. The factor structure (construct) can be determined using factor analysis, which includes exploratory factor analysis (EFA) and confirmatory factor analysis (CFA) [8, 9]. EFA is a data-driven approach that explores the potential factor structure of a measure [10]. Thus, the results of EFA tend to be preliminary and to require confirmation [9–11]. In contrast, CFA is a theory-driven approach that is used to confirm a factor structure [8]. Thus, CFA is a powerful statistical tool for assessing the construct validity [12] of an HRQOL measure.

Two short forms of the commonly used Stroke-Specific Quality of Life (SSQOL) [2] scale have been developed, namely, Wong’s (Hong Kong) version [13] and Post’s (Dutch) version [14] of the 12-item SSQOL. Both versions of the original 12-domain 49-item scale [2, 7] have been analyzed by EFA [15, 16], which identified the same two domains, i.e., a psychosocial domain and a physical domain. However, there are differences in factor structure between the 2 short forms of the 12-item SSQOL. Specifically, only 3 items are the same in both versions. In addition, the two versions classify 3 items into different domains (either the physical or the psychosocial domain) [6, 7]. For example, the items derived from the energy domain are grouped into the physical domain in Wong’s version but the psychosocial domain in Post’s version. The aforementioned differences indicate that the factor structure of the two versions remains unclear, which has limited the utility of both short forms.

Currently, there are two short forms of the 12-item SSQOL available. The construct validity is critical for instrument selections. To our knowledge, no previous studies have compared the underlying two-factor structures of both 12-item SSQOL versions developed by Wong and Post, respectively. It is important for both clinicians and researchers to know which is the better 12-item SSQOL. Thus, a CFA [12, 17] was used to determine which version could be recommended. Specifically, we used CFA to directly examine the construct validity of the physical and psychosocial factor from the 12-item SSQOL using data of the patients with stroke. The examination and confirmation of the construct validity is crucially important and required to an instrument with a subjective latent construct such as the HRQOL. Then, both clinicians and researchers can interpret whether the results obtained from the assessments is accurate or not to represent the patients’ ratings of their HRQOL levels. Therefore, this study used CFA to compare the construct validity of the 2 short forms of the 12-item SSQOL in stroke survivors.

## Materials and methods

### Subjects

We obtained the data from a previous study (see S1 Appendix), which validated 4 versions of stroke-specific HRQOL measures (including 12-domain, 49-item SSQOL) [18]. In that study, the participants were recruited from inpatients admitted to subacute wards and from outpatients at neurology or rehabilitation departments of 5 general hospitals located in the northern and southern regions of Taiwan. The protocol and ethics (for the previous study and this study) were approved by the Institutional Review Board of the Fu-Jen Catholic University and those of the hospitals including Cathay General Hospital where recruitment occurred. Written informed consent was provided by all participants.

The inclusion criteria were as follows: (1) diagnosis of stroke, (2) hemiplegia due to stroke, (3) age over 20 years old, (4) sufficient reading or listening comprehension to complete the self-reported HRQOL measures, and (5) sufficient cognitive ability (with MMSE scores > 22) to follow simple instructions.

### Procedure

After collecting the patients’ baseline information, licensed occupational therapists (OT) administered the National Institutes of Health Stroke Scale (NIHSS) [19], the Mini-Mental State Examination (MMSE) [20], and the Barthel Index (BI) [21]. Patients then completed the SSQOL themselves, and their responses on the 12-item SSQOL were retrieved for the present study.

### Measures

The two short forms of the SSQOL scale [2], i.e., Wong’s [13] and Post’s [14] 12-item SSQOL, were derived from the original SSQOL [2] using EFA. The SSQOL was developed by Williams et al. in 1999 to measure subjective stroke-specific HRQOL. The original SSQOL contained 12 domains and 49 items. Each item was scored on a 5-point Likert-type scale (1–5). The scores based on this 5-point Likert-type scale (1–5) were transformed into a scale from 0–100. Higher scores indicated better levels of patients’ subjective HRQOL. The reliability and validity of the 12-domain SSQOL, including the construct validity, have been demonstrated [2, 18, 22] [7].

Wong’s [13] 12-item SSQOL version has shown satisfactory internal consistency (Cronbach’s alpha values of 0.71–0.90) and criterion validity in patients with subarachnoid hemorrhage (SAH).

Post’s [14] 12-item SSQOL version has shown good internal consistency (Cronbach’s alpha values of 0.78–0.89) and good criterion validity, based on the original 12-domain SSQOL as the gold standard.

Table 1 lists the items and the corresponding domains of the two versions.

**Table 1:** The two short forms of the 12-item SSQOL: Items and their floor/ceiling effect.

	Wong’s version					Post’s version				
Original domains in 12-domain SSQOL	Domain	Item	Mean (SD*)	Floor Effect %	Ceiling Effect %	Domain	Item	Mean(SD*)	Floor Effect %	Ceiling Effect %
Energy	Physical	Item 1: Felt tired all the time	3.0 (1.5)	20.2	24.3	Psychosocial	Item 1: Too tired to do what I wanted to do	3.2 (1.5)	19.8	28.5
Family relation	Physical	Item 2: Physical condition interfered with family life	3.0 (1.5)	21.7	24.7	Psychosocial	Item 2: Felt myself a burden to my family	3.0 (1.6)	21.3	29.3
Language	Psychosocial	Item 3: Having trouble speaking	4.4 (0.9)	1.1	62.0	Physical	Item 3: Need to repeat oneself for others to understand	4.6 (0.8)	8	73.0
Mobility	Physical	Item 4: Having trouble climbing stairs	3.5 (1.5)	18.3	37.6	Physical	Item 4: Need to stop & rest more than you would like when walking or using a wheelchair	3.7 (1.4)	9.5	40.3
Mood	Psychosocial	Item 5: Little confidence in myself	3.6 (1.5)	11.0	42.2	Psychosocial	Item 5: Discouraged about my future	3.6 (1.4)	10.3	42.2
Personality	Psychosocial	Item 6: Impatient with others	3.7 (1.4)	6.8	43.3	Psychosocial	Item 6: Personality has changed	3.2 (1.5)	14.4	33.5
Self care	Physical	Item 7: Need help preparing food?	3.6 (1.7)	25.1	46.0	Physical	Item 7: Need help taking a bath or shower	3.7 (1.5)	15.6	46.4
Social role	Psychosocial	Item 8: Doing hobbies & recreations shorter than expectation	2.7 (1.5)	28.1	24.0	Psychosocial	Item 8: Physical condition interfered with social life	2.8 (1.6)	27.8	26.2
Thinking	Psychosocial	Item 9: Having trouble remembering things	3.5 (1.5)	11.0	37.6	Psychosocial	Item 9: Having trouble remembering things	3.5 (1.5)	11.0	37.6
Upper extremity	Physical	Item 10: Having trouble writing /typing	4.2 (1.2)	4.9	59.7	Physical	Item 10: Having trouble buttoning buttons	4.0 (1.3)	6.8	53.6
Vision	Physical	Item 11: Having trouble seeing a TV well enough to enjoy	4.7 (0.8)	2.3	84.8	Physical	Item 11: Having trouble seeing a TV well enough to enjoy	4.7 (0.8)	2.3	84.8
Work	Physical	Item 12: Having trouble doing daily work around the house	3.5 (1.5)	16.3	40.7	Physical	Item 12: Having trouble doing daily work around the house	3.5 (1.5)	16.3	40.7

*SD: Standard Deviation.

The NIHSS [19] was used to monitor stroke severity. This scale consists of 11 items with a score ranging from 0 to 42. Minor stroke severity is indicated by NIHSS scores ≦3 [23]; mild, 4≦NIHSS≦6 [23, 24]; moderate, 7≦NIHSS≦15 [24]; and severe, NIHSS≧16 [24]. The NIHSS has shown acceptable reliability [25, 26].

The MMSE [20] (with 11 items and a score ranging from 0–30) was used to monitor cognitive dysfunction. The MMSE includes orientation, language, attention, construction, and memory domains. A commonly used cut-point for the MMSE score is 24, with scores less than 24 indicating cognitive impairment. In this study, the criterion was set to MMSE scores > 22, as long as the subjects showed sufficient ability to follow simple instructions.

The BI [21] consists of 10 items and is administered to assess the functional limitations of stroke survivors, indicating different (mild, moderate, and severe) levels of independence in activities of daily living [27]. The possible scores on the BI range from 0–100. The BI has good psychometric properties in patients with stroke [28, 29].

### Data analysis

The CFA [12, 17] was conducted using LISREL 8.70 to model the factor structure of Wong’s version and Post’s version of the 12-item SSQOL. The CFA was used to determine whether the two-factor structure with psychosocial and physical domains was supported in Wong’s version versus Post’s version. The CFA included testing of the models for goodness-of-fit and factor loadings of the 2-factor structure in both versions.

The CFA basically involved the four steps as follows: First, before the model fitting, we have checked the data distribution by examining floor/ceiling effect and Kolmogorov-Smirnov test. The Kolmogorov-Smirnov test was also conducted to test the normality of data. The floor/ceiling effect was analyzed and computed as the percentage of ratings with the lowest/highest point on the scale in each domain. A percentage greater than 20% was considered to indicate a significant floor/ceiling effect [7, 30, 31]. The floor/ceiling effect was likely to cause the distribution of the data skewed [32, 33] and indicated whether the data were normally distributed [34].

Second, we have conducted model fitting of the data. The maximization likelihood (ML) or robust ML was used depending on the normal or non-normal distribution of data. The ML was conducted if the data was normal distributed. Alternatively, the robust ML for fit model was conducted to correct the estimation bias if the data were not normally distributed [7, 35]. After the model fitted using the ML or robust ML method, the goodness-of-fit of each model was assessed with the following fit indices [7, 36–42]: a chi-square/df ratio < 3 suggested good fit; root mean square error of approximation (RMSEA) < 0.08 was considered acceptable and < 0.05, excellent; comparative fit index (CFI) > 0.90 was acceptable and > 0.95, excellent; nonnormative fit index (NNFI) > 0.95 was good; standard root mean square residual (SRMR) < 0.10 was acceptable and < 0.05, good; parsimony normative fit index (PNFI) > 0.50 [43] was acceptable; and higher values PNFI represented an ideal model. Within a set of models for the same data, the model with the minimum Akaike information criterion (AIC) value and consistent AIC (i.e., CAIC) value was considered the best fitting model [7, 44, 45]; i.e., the smaller the AIC and CAIC values were, the better the model fit was. As for the model fitting, a sample size larger than 200 was preferred [46].

Third, we inspected the modification index built-in LISREL to identify if any modification of the model needed. The two domains of the 12-item SSQOL were assumed to be correlated. The two-domain structure of the 12-item SSQOL was tested, by allowing correlations between domains. In the analysis, this step included the conduction of the Pearson’s correlation in LISREL and also the conduction of modification index to examine whether there was the need to add correlations between the factors. We considered to modify model also based on prior research knowledge and clinical experience.

Fourth, we have examined the factor loading of each item to determine if any item was redundant. In this step, the (standardized) factor loadings were analyzed once the goodness-of-fit of the model had been shown. The factor loadings of the items were estimated to represent the correlation between the item and its corresponding factor [47, 48]. We used cutoffs of 0.50 to indicate an acceptable factor loading [49, 50]. This criterion was used to check if there was a need to delete the items with factor loadings lower than it.

## Results

### Characteristics of the participants

The sociodemographic and descriptive information of the participants was shown in Table 2. A total of 263 patients with stroke participated in the study, with a mean age of 59.8 years (SD = 13.0). Most (69.6%) of the participants were men, and most (approximately 80%) had one stroke event. Nearly half (47.1%, n = 124) of the participants were inpatients with a stroke onset within the past three months; the other half (52.1%, n = 137) of the subjects were outpatients. As shown in Table 2, nearly half of the patients had a minor stroke (i.e., NIHSS total score ≦3). On average, the participants showed no cognitive dysfunction (MMSE = 26.2).

**Table 2:** Characteristics of the participants (n = 263).

Variable	Value
Age (year): mean (SD^d^)	59.8 (13.0)
Sex (Male): n (%)	183 (69.6)
Education years: mean (SD^d^)	10.3 (4.2)
Married: n (%)	193 (73.4)
Unemployed status: n (%)	187 (71.1)
Stroke type	
Missing, n (%)	83 (31.6)
Confirmed, n (%)	180 (68.4)
Ischemic: n (%)	125 (69.4)
Hemorrhagic: n (%)	55 (30.6)
The first time of onset: n (%)	216 (82.1)
NIHSS^c^: mean (SD^d^)	4.4 (4.2)
Minor byNIHSS ^c^≦3: n (%)	133 (50.6)
Mild by 6≦NIHSS ^c^ ≦4: n (%)	52 (19.8)
Moderate by 7≦NIHSS ^c^ ≦15: n (%)	73 (27.8)
Severe by NIHSS ^c^ ≧16: n (%)	5 (1.9)
MMSE^b^: mean (SD^d^)	26.19 (3.6)
BI^a^: mean (SD^d^)	79.7 (24.8)
BI^a^ = 100: n (%)	113 (43.0)
Mild by BI^a^ = 95: n (%)	16 (6.1)
Moderate by 61≦BI^a^≦94: n (%)	73 (27.8)
Severe by BI^a^≦60: n (%)	61 (23.2)

BI^a^: Barthel Index.

MMSE^b^: Mini-Mental State Examination.

NIHSS^c^: National Institutes of Health Stroke Scale.

SD^d^: standard deviation.

### The results of the CFA for the factor structures of Wong’s version and Post’s version of the 12-item SSQOL

First, a notable ceiling effect was detected in both versions. The ceiling effects were ranged from 24.0 to 84.8% in Wong’s version, and 26.2.0 to 84.8% in Post’s version (Table 1). That is, there was a significant ceiling effect (> 20%) in all 12 items in both Wong’s and Post’s versions. Each item in Wong’s and Post’s versions showed significant results in the Kolmogorov-Smirnov test (< .001). The hypothesis regarding the normality of data was rejected in the normality test.

Second, the robust ML analysis was conducted because of the non-normality distributions of the data due to the ceiling effect detected. The results of the model fit indices for both versions included the followings (Table 3): (1) The CFI was = 0.97 in Post’s version and was 0.85 in Wong’s version. (2) The parsimonious fitting index includes NNFI and other Goodness-of-fit index (χ^2^/df ratio, RMSEA, SRMR, and PNFI) estimated for the models: In Post’s version, all model fit indices fell within the expected ranges (χ^2^/df ratio = 2.02 < 3.00, RMSEA = 0.05< 0.08, SRMR = 0.06 < 0.10, NNFI = 0.97> 0.95 and CFI = 0.97 > 0.90). In Post’s version, the results of the RMSEA, SRMR, and CFI were close to the criteria indicating an excellent level, better than an acceptable level. In Wong’s version, none of the 5 model fit indices (includingχ^2^/df ratio = 7.77, RMSEA = 0.16, SRMR = 0.11, NNFI = 0.82 and CFI = 0.85) met the criteria. Additionally, the PNFI value was 0.58 in Wong’s version vs. 0.75 in Post’s version (Table 3). (3). The absolute/predictive fit includes the AIC and CAIC values: the model AIC/CAIC values in Post’s version (143.39/257.70) were smaller than the independence AIC/CAIC (1666.50/ 1721.37) and the saturated AIC/CAIC (156.00 / 512.63). In Wong’s version, the model AIC/CAIC (459.57 / 573.87) was smaller than the independence AIC/CAIC (2510.71 / 2565.58) but not the saturated AIC/CAIC (156.00 / 512.63).

**Table 3:** Model fits of Wong’s version and Post’s version of the 12-item SSQOL.

	With Robust ML analysis	
Model fits^1^	Wong’s version	Post’s version
Minimal fit χ^2^	412.11	107.17
*df*	53	53
χ^2^ /*df* ratio	7.77	2.02
RMSEA	0.16	0.05
NNFI	0.82	0.97
SRMR	0.11	0.06
CFI	0.85	0.97
PNFI	0.67	0.76
Independence AIC	2510.71	1666.50
Model AIC	459.57	143.39
Saturated AIC	156.00	156.00
Independence CAIC	2565.58	1721.37
Model CAIC	573.87	257.70
Saturated CAIC	512.63	512.63

*χ*^*2*^:chi-square; *df*: degree of freedom; RMSEA: root mean square error of approximation; CFI: comparative fit index; NNFI: nonnormative fit index; SRMR: standard root mean square residual; PNFI: parsimony normative fit index; AIC: Akaike information criterion; consistent AIC: CAIC.

^1^The criteria of fit indices for acceptable level: a chi-square/df ratio < 3; RMSEA < .08; CFI > 0.90; NNFI > 0.95; SRMR < 0.10; was and > 0.95; the higher values for PNFI/ the smaller the AIC and CAIC values, the better the model fit.

**Fig 1:**
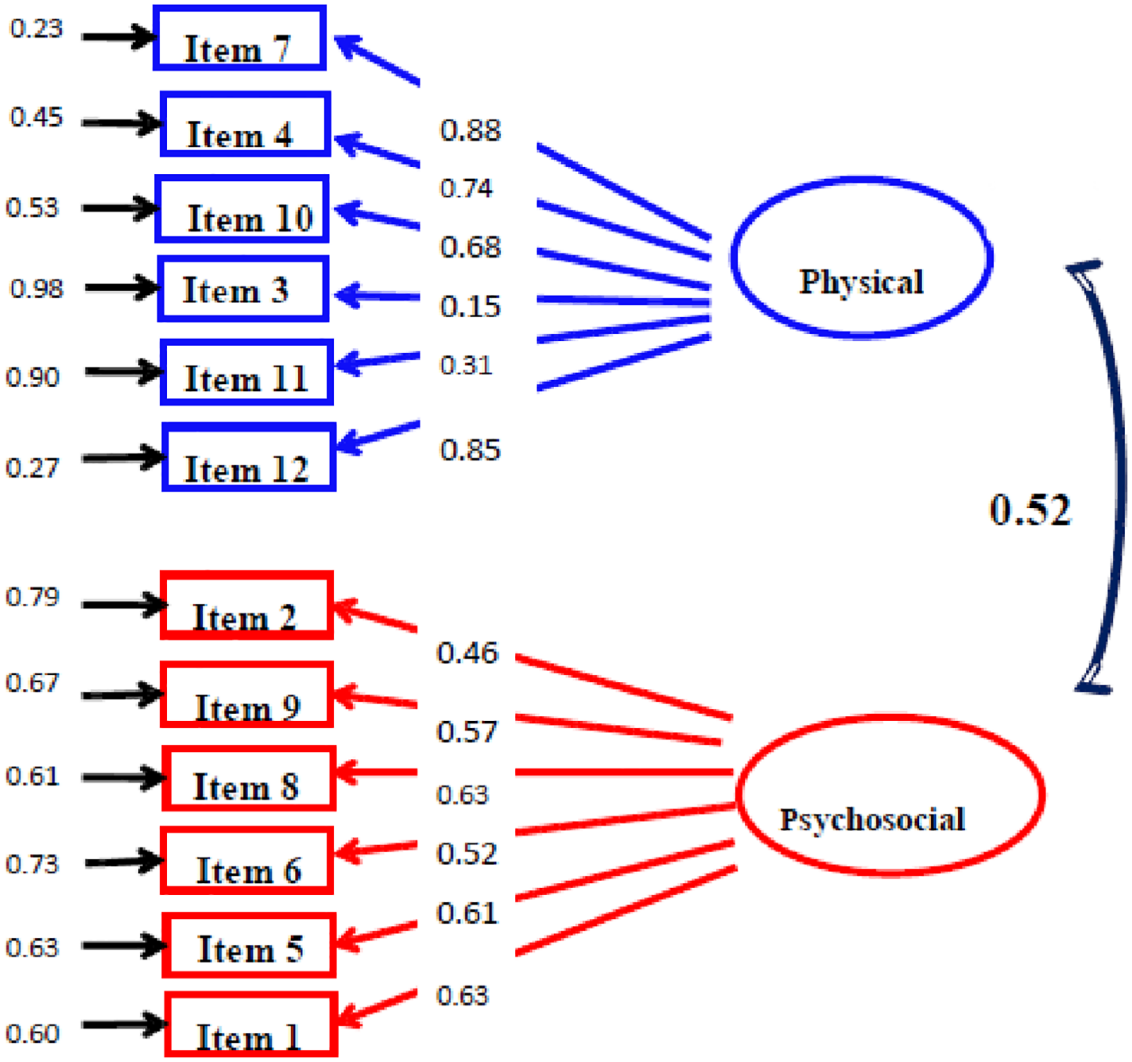
Factor structures of Post’s version of the 12-item SSQOL.

In summary of the model fitting in CFA, Post’s version but not Wong’s version of the 12-item SSQOL showed the satisfactory fitting indices. Therefore, the model of Post’s version was used as our final model of the 12-item SSQOL (Fig 1), without any modification of the model.

Third, because the Post’s model fitting was satisfactory, we did not further inspect the modification index of the model. The Pearson’s correlation coefficient between the physical and psychosocial domains in Post’s version was 0.52 (Fig 1).

Fourth, the (standardized) factor loadings were estimated for the model shown with goodness -of-fit index. The factor loadings could be analyzed in Post’s version of the 12-item SSQOL but not Wong’s version since only one of the five indices met the criterion for analysis of factor loadings. Fig 1 shows the factor loadings of Post’s version of the 12-item SSQOL. For the physical domain, all items but two had acceptable to high factor loadings (0.68 to 0.88). The vision item showed a factor loading of 0.31, and the language item showed a factor loading of 0.15. For the psychosocial domain, all items had factor loadings ranging from 0.46 to 0.63. All items were kept without deletion because the 0.46 was close or borderline to the criteria of 0.5.

## Discussion

Both Wong’s and Post’s versions were validated using CFA. The CFA in these findings was conducted using robust maximum likelihood analysis to adjust for the non-normal distributions, as indicated by the notable ceiling effects (Table 1) detected in both versions. Post’s version of the 12-item SSQOL showed good data-model fit, with all the indices meeting the predetermined criteria. The two domains in Post’s version showed acceptable correlations. Moreover, the item factor loadings in the physical/psychosocial domains were acceptable, although some were low. These findings regarding the factor loadings of the items corresponding to the physical/psychosocial domain explained the data well. Overall, the current CFA findings supported Post’s version over Wong’s version.

In addition to the borderline factor loading (0.46) of the family item, two items in Post’s version had notably low factor loadings. The language item (i.e., “Did you have to repeat yourself so others could understand you?”) had a low factor loading of 0.15. Additionally, the vision item (i.e., “whether having trouble seeing a TV well enough to enjoy?”) had a low factor loading of 0.31. These low factor loadings may be because this particular sample encountered little difficulty with repeating language and watching TV. Seventy-three percent of the participants (Table 1) scored 5 points on the language item, indicating no difficulty at all. Additionally, the mean score of this item was 4.6 (SD = 0.8). These subjects may have had few difficulties with sight or language, as these factors may be prerequisites for completing the self-rated HRQOL questionnaires. However, we decided to keep this item because a substantial proportion of patients can still experience language difficulties such as aphasia after onset of stroke. Furthermore, the item showed good model fit. Provided that the language and vision items are retained, the 2-factor structure of Post’s version of the 12-item SSQOL is recommended. These findings indicate that the construct validity of Post’s version of the 12-item SSQOL is well supported for measuring two-dimensional HRQOL in patients with stroke.

Moreover, the preference for Post’s version over Wong’s version is also supported by the model comparison results. Specifically, Post’s version showed a better model fit but not Wong’s version. The model AIC/CAIC values in Post’s version were smaller than the independence AIC and the saturated AIC/CAIC. However, in Wong’s version the model AIC/CAIC was smaller than the independence AIC but not the saturated AIC/CAIC. In summary, Post’s version of the 12-item SSQOL is recommended. Wong’s version showed poor model fit, with all 6 indices of goodness of fit not meeting the criteria. Thus, the two-factor structure of Wong’s version was rejected.

Our findings of the model fit supported the 2-factor structure of Post’s version but not for that of Wong’s version. These may be due to the differences of the item constitution in their structures. The constitution of the items in Post’s version may better meet the psychological or social concerns of the patients. For example, the “family relation” item is generally expected to have higher relation with “psychosocial” domain, rather than higher relation with “physical domain”. This may be the main reason to explain why the Wong’s version was not supported but the Post’ version in which the “family relation” item in Post’s version grouped into psychosocial domain whereas the “family relation” item in Wong’s version was grouped into “physical domain”. In detail, in Post’s version the item “Felt myself a burden to my family” (Table 1) presenting the emphasis on psychosocial aspect, while the “family relation” item in Wong’s version was “Physical condition interfered with family life” (Table 1) showing the emphasis on physical aspect. As a result, the item constitution of the 2 domains in Post’s version can be better used to reflect the patients’ HRQOL after stroke. In summary, the current findings do support the usage of the Post’s version, but not Wong’s version, of the 12-item SSQOL.

Notably, the short-form 12-item SSQOL cannot be used to replace the original 12-domain SSQOL. In particular, the original SSQOL can be used as an outcome measure to assess a patient’s common concerns and can contribute to a better understanding of the patient based on the 12 domains in the SSQOL. The main advantage of the original 12-domain SSQOL is that it provides a more detailed profile (subscale/domain scores) than the 12-item SSQOL (with only physical/psychosocial subtotal scores). However, the two domains of the short forms of the 12-item SSQOL can indicate patients’ core concerns and needs, which can be useful for helping clinicians and researchers provide effect patient management. Moreover, the multiple domains inevitably require the completion of numerous items, which can be time consuming, thus limiting the feasibility of the full version for regular use. Fortunately, these difficulties can be reduced by using the short forms of the SSQOL. These short forms have the advantage of decreasing patients’ testing burden and shortening the completion time. Thus, it is practical to use the 12-item SSQOL as a quick and feasible outcome measurement for stroke survivors.

This study has the following limitations. First, the comparisons between the 2 short forms were based on a secondary analysis of data collected primarily to validate the original 12-domain SSQOL, not directly to compare Wong’s and Post’s versions of the 12-item SSQOL. Although the data were no longer primary and were used for secondary analysis, the use of the same data allowed the comparison of Post’s and Wong’s 12-item-SSQOLs simultaneously on the same basis of the data employed for testing 4 versions of the SSQOL and SIS validated and published earlier [18]. Further validation studies with a prospective design that directly administer Wong’s and Post’s versions of the 12-item SSQOL are encouraged. Second, the sample recruited in this study tended to have a mild level of stroke and functional limitations; specifically, 70.4% of the participants had minor to mild stroke severity according to the NIHSS. This mild level of stroke severity led to the prominent ceiling effects (larger than 20%) in both versions, as shown in previous findings [7] (Table 1). Accordingly, the current findings may not be generalizable to patients with severe stroke. Further validations that include severe stroke survivors are recommended.

The content validity and face validity are also important for clinicians to consider for clinical applicability. Generally speaking, the content validity and face validity are examined during the stage of the instrument development and translation. It is noted that all the versions of SSQOL mentioned in this study were translated versions. However, the content validity and face validity of these translated versions are not reported, which might have affected our results and interpretations. At this stage, prospective users should consider the content validity and face validity of the short forms by themselves. In addition, other psychometric properties of the scales, such as test-retest reliability and responsiveness, need to be determined. Finally, calculating the minimally important difference would enhance the utility of the short-form 12-item SSQOL.

The sample size in the current study is acceptable for the conduction of the CFA. The sample size of 263 in this study had an acceptable ratio of 10.5 participants to 1 parameter estimated (25 parameters in total). In other words, the sample size met the need of CFA; although there is no exact rule for the number of participants needed, 10 per estimated parameter appears to be the general consensus [42]. Also, our sample size of 263 also fell between the 200 required for testing a theoretical model and the 300 required for testing a population model [46].

The recruitment was originally conducted by using convenience sampling, so the representativeness of the sample was limited due to the lack of randomness. Although the data is no longer primary and is used for secondary analysis, the use of the same data enables the comparison of both 2 short forms of the Post’ and Wong’s 12-item SSQOL simultaneously comparable on the same basis of the data employed for testing 4 versions of the SSQOL and SIS validated and published earlier [18].

Overall, the CFA supported the two-factor structure in Post’s version, which showed sound construct validity and used 12 items only. Therefore, Post’s version of the 12-item SSQOL may save time in the assessment of HRQOL in clinical settings.

## Supporting information

S1 Appendix (PDF)
