# Supplementary material for: Comparison of construct validity of two short forms of Stroke-Specific Quality of Life scale
Source: PLoS One. 2017 Dec 6;12(12):e0188478. doi: 10.1371/journal.pone.0188478 (PMC5718408; doi:10.1371/journal.pone.0188478)
Supplement: S1 Appendix — (PDF) [file pone.0188478.s001.pdf]

Clinical Rehabilitation
http://cre.sagepub.com/

Psychometric comparisons of four disease-specific health-related quality of life measures for stroke survivors

Chia-Yeh Chou, Yu-Chih Ou and Tsuey-Ru Chiang

Clin Rehabil published online 28 October 2014
DOI: 10.1177/0269215514555137

The online version of this article can be found at:
http://cre.sagepub.com/content/early/2014/10/20/0269215514555137

Published by:
SAGE
http://www.sagepublications.com

Additional services and information for *Clinical Rehabilitation* can be found at:

Email Alerts: http://cre.sagepub.com/cgi/alerts

Subscriptions: http://cre.sagepub.com/subscriptions

Reprints: http://www.sagepub.com/journalsReprints.nav

Permissions: http://www.sagepub.com/journalsPermissions.nav

>> OnlineFirst Version of Record - Oct 28, 2014

What is This?

Downloaded from cre.sagepub.com at Fu Jen Catholic University, on October 29, 2014

Article

CLINICAL REHABILITATION

Clinical Rehabilitation
1–14
© The Author(s) 2014
Reprints and permissions:
sagepub.co.uk/journalsPermissions.nav
DOI: 10.1177/0269215514555137
cre.sagepub.com
SAGE

Psychometric comparisons of four disease-specific health-related quality of life measures for stroke survivors

Chia-Yeh Chou[1], **Yu-Chih Ou**[2] **and Tsuey-Ru Chiang**[3]

Abstract

Objective: To examine psychometric properties of four stroke-specific health-related quality of life (HRQoL) measures, including original Stroke-Specific Quality of Life Scale (12-domain SSQoL), modified 8-domain SSQoL, Stroke Impact Scale (SIS 3.0), and modified SIS-16 focused on physical domains.
Design and Setting: Prospective repeated measures study conducted in rehabilitation and wards in hospitals.
Subjects: Study cohort was recruited with 263 patients in the first administration and 121 in the second administration, an average of two weeks later. To investigate discriminant validity, the same number of patients (i.e., 52) was grouped for each of 3 levels of stroke severity.
Main measures: Outcome measures, including National Institutes of Health Stroke Scale, Mini-Mental State Examination, and Barthel Index. Patients completed HRQoL self-reports.
Results: Domains of four measures showed (1) good reliability, except 12-domainSSQoL family roles (Cronbach's α = 0.68) and personality domains (Cronbach's α = 0.65) and SIS 3.0 social participation (ICC=0.67) domain; (2) acceptable precision, except 12-domain SSQoL family role domain and SIS 3.0 social participation domain; (3) good convergent validity, except 12-domain SSQoL/8-domain SSQoL vision domain (r = 0.19), (4) good discriminant validity, except 12-domain SSQoL and 8-domain SSQoL thinking domains (*P* = 0.365); and (5) acceptable floor effects and strong ceiling effects. The 12-domain SSQoL and 8-domain SSQoL met scaling assumptions better than SIS 3.0 and SIS-16.
Conclusions: Four measures showed acceptable psychometric properties with some domains slightly less satisfactory. Overall, use of 8-domain SSQoL and SIS 3.0 are feasible for clinical practice to monitor HRQoL of stroke survivors.

Keywords

Quality of life, stroke, outcome measures, psychometric properties, disease-specific measures

Received: 3 June 2014; accepted: 20 September 2014

[1]Department of Occupational Therapy, College of Medicine, Fu-Jen Catholic University, Taiwan
[2]Department of Rehabilitation, Mackay Memorial Hospital and Mackay Medicine, Nursing and Management College, Taiwan
[3]Department of Neurology, Cathay General Hospital and Fu-Jen Catholic University, Taiwan

Corresponding author:
Chia-Yeh Chou, Department of Occupational Therapy, College of Medicine, Fu-Jen Catholic University, No. 510 Zhong Zheng Road, Xinzhuang Dist., New Taipei City, 24205 Taiwan.
Email: 068213@mail.fju.edu.tw

Downloaded from cre.sagepub.com at Fu Jen Catholic University, on October 29, 2014

Introduction

Stroke often leads to high mortality or disability and has a multidimensional impact on the daily living of patients. The impact of stroke can be assessed by multiple subscales of health-related quality of life measures. The best choices to investigate health-related quality of life levels of stroke patients are disease-specific health-related quality of life instruments.

The disease-specific health-related quality of life measures are designed on the basis of patients' input and disease characteristics. Two main disease-specific health-related quality of life measures commonly used for stroke survivors are the Stroke-Specific Quality of Life Scale[1] and the Stroke Impact Scale.[2] In the past decade, these two measures have evolved into a series of stroke-specific health-related quality of life measures, including: (1) the original Stroke-Specific Quality of Life Scale with 12 domains (12-domain Stroke-Specific Quality of Life Scale),[1] (2) the shortened Stroke-Specific Quality of Life Scale with 8 domains (8-domain Stroke-Specific Quality of Life Scale),[3] (3) the Stroke Impact Scale 3.0[4, 5] modified from the original Stroke Impact Scale 2.0,[2] and (4) the Stroke Impact Scale-16,[6] which basically emphasizes the physical domain. In addition, the four physically relevant domains (i.e., self care, mobility, upper extremity function, work) of the 12-domain Stroke-Specific Quality of Life Scale have been validated and converged as "activities" [3] in the 8-domain Stroke-Specific Quality of Life Scale. Similarly, a domain named "composite physical domain" [4] represents the combination of the strength, hand function, ADL/ Instrumental ADL activities, and mobility domains in the Stroke Impact Scale 3.0. These four health-related quality of life measures have different advantages. The Stroke Impact Scale 3.0 and Stroke Impact Scale-16 were designed originally to investigate the disease impact of stroke, rather than actual health-related quality of life levels, and both have been validated by Rasch analysis. In contrast, the 12-domain Stroke-Specific Quality of Life Scale and 8-domain Stroke-Specific Quality of Life Scale were designed originally to assess health-related quality of life levels of stroke survivors. However, the 12-domain Stroke-Specific Quality of Life Scale and 8-domain Stroke-Specific Quality of Life Scale have not been validated by Rasch analysis. Exploratory factor analysis has been used to confirm the unidimensionality of only the domains of 12-domain Stroke-Specific Quality of Life Scale.[1] Different versions of the Stroke-Specific Quality of Life Scale[1, 3] and Stroke Impact Scale[2, 4–6] have been validated and recommended[7, 8].

Although the health-related quality of life measures are used to assess global outcome, there are currently few or no comparative studies allowing a clinician or a researcher to select appropriate measures. In other words, no study has ever comprehensively compared all of the versions of these two disease-specific health-related quality of life measures simultaneously with the same study design and the same study sample. The factor structure of the 12-domain Stroke-Specific Quality of Life Scale has been supported but not the 8-domain Stroke-Specific Quality of Life Scale compared.[7] When being compared with the SIS 3.0, the 12-domain Stroke-Specific Quality of Life Scale become less satisfactory psychometric properties than the Stroke Impact Scale 3.0.[8] This prospective study was the first to compare the psychometric properties of the 12-domain Stroke-Specific Quality of Life Scale, 8-domain Stroke-Specific Quality of Life Scale (with "activities"), Stroke Impact Scale 3.0 (with "composite physical domain"), and Stroke Impact Scale-16. Specifically, the reliabilities of the four stroke-specific measures were to be investigated, including the acceptability, internal consistency, test-retest reliability, and precision i.e., absolute reliability. Also, the convergent validity and discriminant validity of the four measures were to be examined due to the lack of the gold standard. The hypothesis was that the 8-domain Stroke-Specific Quality of Life Scale and Stroke Impact Scale 3.0 would show better psychometric properties than the others.

Subjects and methods

Subjects

By convenient sampling, participants were recruited from patients admitted to acute wards or rehabilitation centers, or from those making clinical visits, mainly in the departments of neurology or rehabilitation of five general hospitals in the north and south

Downloaded from cre.sagepub.com at Fu Jen Catholic University, on October 29, 2014

area of Taiwan. The recruitment was supported by the research grants from Taiwan National Science Council and lasted from August 2008 to June 2010. Inclusion criteria were: (1) diagnosis of stroke based on DSM-IV (1994), (2) hemiplegia due to stroke, (3) age over 18 years old, (4) sufficient reading or listening comprehension ability to complete the self-reported health-related quality of life measures, and (5) sufficient cognitive ability to follow simple instructions.

Procedure

The protocol and ethics were approved by the Institutional Review Board of Fu-Jen Catholic University and by the general hospitals where recruitment occurred. All participants provided informed consent. After the baseline information was collected, licensed occupational therapists (OT) trained in the measures administered the Mini-Mental State Examination, National Institutes of Health Stroke Scale, and Barthel Index to the subjects at the same time, rather than the information taken from the hospital records. Consecutively, the subjects completed self-reports of the 12-domain Stroke-Specific Quality of Life Scale, Stroke Impact Scale 3.0, and WHQOoL-BREF Taiwan version. These measures were conducted in a random order of administration within three days to minimize the effects of spontaneous recovery or other factors potentially influential to patients' health-related quality of life levels. The subjects willing to receive evaluations for follow-up completed the second administration of the health-related quality of life measures an average of two weeks later. All the subjects and therapists were blinded to the purpose of the study and the score computations during the study period.

Measures

The 12-domain Stroke-Specific Quality of Life Scale,[1] and 8-domain Stroke-Specific Quality of Life Scale,[3] were used to measure subjective stroke-specific health-related quality of life levels. The 12-domain Stroke-Specific Quality of Life Scale[1, 7] (originally 12 domains, 49 items) was developed by Williams et al., in 1999 and was later shortened to the 8-domain Stroke-Specific Quality of Life Scale[3] (8 domains, 47 items) by conducting factor analysis. Only three domains, i.e., personality, thinking, and vision, retained the same items from the 12-domain Stroke-Specific Quality of Life Scale. The scores of both the 12-domain Stroke-Specific Quality of Life Scale and 8-domain Stroke-Specific Quality of Life Scale were rated on a 5-point Likert-like scale and were transformed to 0-100 scaling. Higher scores indicate better levels of the patients' subjective health-related quality of life status. The 12-domain Stroke-Specific Quality of Life Scale and 8-domain Stroke-Specific Quality of Life Scale have been validated with psychometric properties in Taiwan.[7,8]

The Stroke Impact Scale 3.0,[4, 5] and Stroke Impact Scale-16[6] were also used to measure subjective stroke-specific health-related quality of life levels. The Stroke Impact Scale 3.0[4, 5] (8 domains, 59 items) was derived by Rasch analysis from the original Stroke Impact Scale 2.0[2] (8 domains, 64 items) developed by Duncan et al., in 1999. By Rasch analysis, the Stroke Impact Scale-16[6] (with 16 items) has been derived and validated from the Stroke Impact Scale 3.0 composite physical domain (with 28 items). The Stroke Impact Scale-16 focuses on health-related quality of life levels related to physical function, since the 16 items mainly come from the original two Stroke Impact Scale 3.0 domains: (1) ADL/ Instrumental ADL activities and (2) mobility.[6] The scores of both the Stroke Impact Scale 3.0 and Stroke Impact Scale-16 were rated on a 5-point Likert-like scale. The Stroke Impact Scale 3.0 and Stroke Impact Scale-16 scores were transformed to 0-100 scaling. Higher scores indicate better levels of the patients' subjective health-related quality of life status. The Stroke Impact Scale 3.0 has been validated and supported with acceptable psychometric properties in Taiwan.[8]

To validate the disease-specific health-related quality of life measures, the generic and widely-used World Health Organization Quality of Life-BREF Taiwan version[9, 10] was used. The World Health Organization Quality of Life-BREF [9] has 26 items in 4 domains. The scores of the World Health Organization Quality of Life-BREF were rated on a 5-point Likert-like scale. The World Health Organization Quality of Life-BREF scores were

Downloaded from cre.sagepub.com at Fu Jen Catholic University, on October 29, 2014

transformed to 0-100 scaling. Higher scores indicate better levels of the patients' subjective health-related quality of life status. For international comparison, two additional items (i.e., self-esteem and eating) adapted for the World Health Organization Quality of Life-BREF Taiwan version[10] were excluded from this study. No use of the total score for the four subscales is suggested.[10]

The National Institutes of Health Stroke Scale[11] was used to monitor the stroke severity of stroke patients. The National Institutes of Health Stroke Scale[11] consists of 11 items. Higher scores indicate more severe levels of the patients' disease severity status. Total score ranges from 0 to 42. Severity of stroke is determined by cut-points: minor level is indicated by National Institutes of Health Stroke Scale scores ≦3;[12] mild level is indicated by 4≦National Institutes of Health Stroke Scale≦6;[12,13] moderate level is indicated by 7≦National Institutes of Health Stroke Scale≦15;[13] and severe level is indicated by National Institutes of Health Stroke Scale ≦16.[13] The National Institutes of Health Stroke Scale has been shown to have acceptable reliability.[14,15]

The Mini-Mental State Examination[16] was used to monitor the cognitive dysfunction of stroke patients. The Mini-Mental State Examination[16] consists of 11 items in the domains of orientation, language, attention, construction, and memory. Total score ranges from 0 to 30. Higher scores indicate better levels of the patients' cognitive status. A score of less than 24 is generally used to indicate cognitive impairment. Additionally, in this study, patients with Mini-Mental State Examination scores of slightly less than 24 were not excluded if they still showed comprehensive ability to complete the health-related quality of life self reports.

The Barthel Index[17] was used to monitor the functional limitations of stroke patients. The Barthel Index[17] has 10 items, and the scores indicate mild, moderate, and severe levels of independence in activities of daily living.[18] The Barthel Index scores were transformed to 0-100 scaling. Higher scores indicate better levels of the patients' independent status. The Barthel Index has been shown to have good psychometric properties in stroke survivors.[19, 20]

Data analysis

All statistical analyses were computed by SPSS 18.0. The statistical significance level was set at $P<$ 0.05 (2-sided). Acceptability was investigated as follows. The mean score should be around the midpoint of the domain score.[21] Biased score distributions (ex: mean score deviated far from midpoint) indicated only a limited range of the patients assessed, rather than involving all variations of patient characteristics. Ceiling/floor effects were estimated by the percentage of participants with maximum/minimum scores reported for each domain.[22] A percentage higher than 20% indicated significant ceiling/floor effects.[22] To examine the score scaling assumption of whether the items were correctively grouped into domains, an item-domain correlation corrected with values of 0.40 or higher was considered acceptable.[23]

Test-retest reliability was analyzed by calculating intraclass correlation coefficients. To ensure that the status remained stable between the two administrations, a non-parametric Wilcoxon signed rank test was conducted to find significant differences in health-related quality of life scores rated in the first- and second administrations. A value at least 0.70 was suggested for acceptable test-retest reliability[24] and acceptable internal consistency at the group level.[24] The internal consistency of each domain was tested by calculating its Cronbach's alpha (α) coefficient.[25]

Standard error of measurement was the absolute reliability to quantify the actual size of random variability between the measurements obtained from two test administrations and was referred to as Standard error of measurement=√ within subject variance or √Mean square error.[26] Moreover, the standard error of measurement was often calculated as standard error of measurement=standard deviation (of all test-retest scores) × √(1-Intra-class correlation coefficients).[27] Acceptable standard error of measurement was required to meet standard error of measurement ≦ standard deviation /2.[28] The amount of variability (obtained from two test administrations) smaller than the value of standard error of measurement represented acceptable random measurement error and thus was indicative of reliability.

Downloaded from cre.sagepub.com at Fu Jen Catholic University, on October 29, 2014

The smallest real difference refers to the smallest change indicative of a real improvement for a single subject. The smallest real difference was computed to show whether a change value was indicative of real change at the 95% confidence level, i.e., the smallest real difference was calculated by 1.96 × standard error of measurement × √2.[26, 27] In this formula, the greater the value of the intra-class correlation coefficients, the smaller the standard error of measurement or smallest real difference, and thus the better the precision i.e., absolute reliability indicated.[26] In other words, a change score equal to or exceeding this threshold is true and reliable and not just measurement error. Convergent validity was checked if there were moderate (r = 0.30 to 0.59) to high (r ≧ 0.60)[1,5,21] correlations between individual domains of the 12-domain Stroke-Specific Quality of Life Scale, 8-domain Stroke-Specific Quality of Life Scale, Stroke Impact Scale 3.0, and Stroke Impact Scale-16 with similar constructs in the World Health Organization Quality of Life-BREF Taiwan version, National Institutes of Health Stroke Scale, or Mini-Mental State Examination. Also, a fair degree of association was indicated if correlation coefficients reached 0.25.[29] Spearman rank correlation coefficient (ρ) was used to investigate the convergent validity.

Known-group methodology[30] was used to examine the discriminant validity and to identify significant differences in health-related quality of life scores among subgroups. These subgroups were grouped by the severity of stroke disease which was determined by the assessments of the National Institutes of Health Stroke Scale. The ANOVA was used to examine if there was the significant differences of health-related quality of life scores among the subgroups.

Results

A total of 263 participants were recruited. Just under half (47%, *n*=124) of the participants were inpatients with onsets within three months recruited from hospital wards, and just over half (52%, *n*=137) of the subjects were outpatients recruited from clinical visits or rehabilitation centers. Most of the stroke patients were receiving or had received rehabilitation (70 %, *n*=183). Nearly half of the patients (121) completed the second administration of the health-related quality of life measures. Most (97%, *n* =255) of the patients who completed the second administration were outpatients, and 90% (n=237) of these patients were receiving rehabilitation. The patients who completed the first and second administrations had similar sociodemographic and clinical features (Table 1). Nearly half of the patients presented mild restriction of daily living independence.

The psychometric results are presented in Tables 2–5. The floor effects were mostly acceptable in four health-related quality of life measures. More domains were reported with ceiling effects over 20%. The ceiling effects were 10-72 % in 12-domain Stroke-Specific Quality of Life Scale; 8-33% in 8-domain Stroke-Specific Quality of Life Scale; 6-60 % in Stroke Impact Scale 3.0; and 14% in Stroke Impact Scale-16. Only some domains had borderline Cronbach's alpha and Intra-class correlation coefficients scores. The Cronbach's alpha was 0.65-0.94 in the 12-domain Stroke-Specific Quality of Life Scale; 0.77-0.96 in the 8-domain Stroke-Specific Quality of Life Scale; 0.80-0.98 in Stroke Impact Scale 3.0; and 0.94 in Stroke Impact Scale-16. The Intra-class correlation coefficients value was 0.70-0.91 in the 12-domain Stroke-Specific Quality of Life Scale; 0.79-0.93 in the 8-domain Stroke-Specific Quality of Life Scale; 0.67-0.97 in Stroke Impact Scale 3.0; and 0.95 in Stroke Impact Scale-16. The Smallest real difference values of ranged from 17.8 (vision) to 44.0 (family roles) for the 12-domain Stroke-Specific Quality of Life Scale, from 17.2 (Activities) to 37.5 (thinking) for the 8-domain Stroke-Specific Quality of Life Scale, and from 8.4 (ADL/Instrumental ADL activities) to 46.3 (emotion) for the Stroke Impact Scale 3.0. The Smallest real difference value of the Stroke Impact Scale-16 was 13.2. Most domains showed at least fair level of correlation coefficients, and only some domains such as vision domain were found small correlation coefficients. Only the thinking domain of the 12-domain Stroke-Specific Quality of Life Scale/8-domain Stroke-Specific Quality of Life Scale did not reach significant level in the ANOVA analysis.

Downloaded from cre.sagepub.com at Fu Jen Catholic University, on October 29, 2014

Table 1. Patient characteristics at baseline and re-test.

Characteristic	Baseline		Re-test		*t* test or χ2 test
	N=263		*N* =121		
Male (*N*, %)	183	70	79	65.0	χ2 =0.70
Age years (mean, SD)	59.8	13.0	59.5	11.0	*t* =0.21
Age≦50 (*N*, %)	59	22	24	20	
51≦Age≦60 (*N*, %)	74	28	38	31	
61≦Age≦70 (*N*, %)	77	29	41	34	
Age≧71(*N*, %)	53	20	18	15	
Education years (mean, SD)	10.3	4.2	9.9	4.1	*t* =9.67
Elementary school and none (*N*, %)	84	32	44	36	
Junior high school (*N*, %)	43	16	20	17	
Senior high school (*N*, %)	72	27	32	26	
College and higher (*N*, %)	64	24	25	21	
Married (*N*, %)	193	73	90	74	χ2 =0.08
Unemployed status (*N*, %)	187	71	104	86	χ2 =9.95**
Stroke type					χ2 =0.28
Ischemic (*N*, %)	125	48	71	59	
Hemorrhagic (*N*,%)	55	21	27	22	
Number of onsets					χ2 =0.39
One	216	82	97	81	
Two	42	16	20	17	
NIHSS total score (mean, SD)	4.4	4.2	4.2	3.5	*t* = 0.48
Minor: NIHSS≦3 (*N*, %)	133	51	59	48	
Mild: 6≦NIHSS≦4 (*N*, %)	52	20	30	25	
Moderate: 7≦NIHSS≦15 (*N*, %)	73	28	32	26	
Severe: NIHSS≧16 (*N*, %)	5	2	0	0	
MMSE total scores (mean, SD)	26.19	3.6	26.7	3.2	*t* = –1.45
BI total scores (mean, SD)	79.7	24.8	87.6	19.0	*t* = –3.11**
Mild: 95≦BI≦100 (*N*, %)	129	49	74	61	
Moderate: 61≦BI≦94 (*N*, %)	73	28	34	28	
Severe: BI≦60 (*N*, %)	61	23	13	11	

BI: Barthel Index; MMSE: Mini-Mental State Examination; NIHSS: National Institutes of Health Stroke Scale; **P* <0.05; ***P* <0.01.

Discussion

In this prospective study, the four closely relevant health-related quality of life measures showed acceptable reliability, precision, convergent validity, and discriminant validity, although their acceptability is less than satisfactory. The participants recruited for the validations in the first test administration were both inpatients and outpatients recruited from northern and southern areas of Taiwan. The majority of patients willing to receive the second administration was outpatients, whose disease severity can be less severe and more likely to have social participation and thus possibly makes the retest achievable. The acceptability, internal consistency, and convergent validity were validated by the sample, which had even distributions of age. To test the test-retest reliability and precision (i.e., absolute reliability), the similarities in the sample characteristics of the first and the second administration were highly similar, with the one difference being the sample size. However, the sample size was controlled among the groups of

Downloaded from cre.sagepub.com at Fu Jen Catholic University, on October 29, 2014

Table 2. Acceptability, reliability, and precision of 12-domain SSQoL vs. 8-domain SSQoL.

Domains	No. of item	Mean±SD	Floor effect %	Ceiling effect %	Item-domain correlation corrected	Cronbach's alpha	Wilcoxon Z value	ICC	SEM	SRD
		N=263					*N*=121			
**12-domain SSQoL**										
Energy	3	51.0±34.0	13	18	0.75–0.78	0.88	−0.43	0.80	15.4	42.6
Family roles	3	56.7±29.0	3	16	0.36–0.60	**0.68**	−0.33	0.70	15.9	44.0
Language	5	87.4±17.5	1	**42**	0.67–0.80	0.89	−0.68	0.78	7.0	19.4
Mobility	6	69.9±30.3	5	**23**	0.81–0.85	0.94	0.12	0.88	8.4	23.2
Mood	5	67.2±26.1	2	19	0.42–0.66	0.79	1.02	0.75	13.0	35.8
Personality†	3	62.5±27.8	2	22	0.28–0.59	**0.65**	−0.09	0.79	13.2	36.6
Self–care	5	74.0±28.2	1	**34**	0.46–0.83	0.87	−1.16	0.91	7.8	21.6
Social roles	5	45.9±29.3	8	10	0.54–0.67	0.83	−0.50	0.81	12.8	35.5
Thinking†	3	63.5±31.4	5	**24**	0.56–0.76	0.82	0.86	0.81	13.5	37.5
UE function	5	75.3±26.5	1	**30**	0.44–0.79	0.86	0.59	0.85	9.5	26.3
Vision†	3	91.3±18.4	1	72	0.53–0.78	0.84	1.46	0.84	6.4	17.8
Work	3	61.5±34.1	11	**24**	0.66–0.79	0.86	−0.27	0.87	12.0	33.1
Total score	49	67.2±18.6	—	—	0.31–0.73	0.95	−0.06	0.91	6.4	17.8
**8–domain SSQoL**										
Energy	4	56.7±29.6	4	14	0.39–0.75	0.82	−0.12	0.79	13.4	37.2
Activities	18	69.5±26.4	1	8	0.42–0.85	0.96	−0.16	0.93	6.2	17.2
Language	6	85.9±17.3	1	**33**	0.40–0.77	0.87	−0.39	0.81	6.3	17.5
Mood	4	65.5±27.4	2	**22**	0.51–0.61	0.77	0.92	0.79	12.9	35.8
Social roles	6	50.1±27.6	3	9	0.46–0.67	0.83	−0.40	0.79	12.5	34.7
Total score	47	68.3±18.7	—	—	0.31–0.73	0.95	0.14	0.91	5.4	14.9

†:The same domain is included in the 8–domain SSQoL. Bold numbers indicate results without meeting the criteria. ICC: Intra–class correlation coefficients; SEM: standard error of measurement; SRD: smallest real difference; SSQoL: Stroke–Specific Quality of Life Scale; **P*<0.05; ***P*<0.01

Downloaded from cre.sagepub.com at Fu Jen Catholic University, on October 29, 2014

Table 3. Acceptability, reliability, and precision of SIS 3.0 vs. SIS–16.

Domains	No of item	Mean±SD	Floor effect %	Ceiling effect %	Item–domain correlation corrected	Cronbach's alpha	Wilcoxon Z value	ICC	SEM	SRD
		N=263					*N*=121			
**SIS 3.0**										
Strength	4	48.8±30.7	10	11	0.79–0.83	0.92	−0.08	0.82	10.9	30.1
Hand function	7	52.2±41.2	**26**	**24**	0.90–0.96	0.98	0.27	0.96	3.7	10.3
Mobility	9	69.2±29.2	1	19	0.52–0.90	0.95	−0.53	0.93	5.4	15.0
ADL/IADL	7	72.2±25.6	1	**22**	0.34–0.87	0.91	−1.15	0.95	3.0	8.4
Memory and thinking	10	85.9±18.0	1	**37**	0.55–0.78	0.90	0.02	0.79	10.3	28.5
Communication	9	92.1±14.7	1	**60**	0.63–0.77	0.90	0.75	0.78	11.7	32.4
Emotion	5	68.5±19.6	1	6	0.29–0.62	0.80	−2.14*	0.83	16.7	46.3
Social participation	8	62.6±28.0	2	16	0.52–0.75	0.89	0.05	**0.67**	14.9	41.2
Total score	59	68.9±19.3	—	—	0.17–0.79	0.96	−0.58	0.94	4.3	11.8
Composite physical	28	64.3±26.6	1	8	0.28–0.85	0.97	−0.69	0.97	4.3	12.0
**SIS–16**	16	71.8±25.1	1	14	0.33–0.86	0.94	−0.73	0.95	4.8	13.2

Bold numbers indicate results without meeting the criteria. Bold numbers indicate results without meeting the criteria. ICC: intra–class correlation coefficients; SEM: standard error of measurement; SIS: Stroke Impact Scale; SRD: smallest real difference; **P*<0.05; ** *P*<0.01

Downloaded from cre.sagepub.com at Fu Jen Catholic University, on October 29, 2014

Table 4. Convergent and discriminant validity of 12–domain SSQoL vs. 8–domain SSQoL.

Domains	WHOQoL–BREF				NIHSS	MMSE	NIHSS			
	Physical	Psychological	Social	Environment			Minor	Mild	Moderate	*P value*
	N=263						*N*=52	N=52	*N*=52	
**12–domain SSQoL**										
Energy	**0.47****	0.46**	0.15*	0.31**	−0.23**	0.25**	61.7	40.7	43.4	.005
Family roles	0.50**	0.49**	**0.17****	0.41**	−0.44**	0.24**	72.9	48.7	38.0	.000
Language	0.25**	0.29**	0.18**	0.24**	**−0.27****	**0.20****	91.9	82.4	84.4	.018
Mobility	**0.64****	0.35**	0.24**	0.35**	−0.52**	0.20**	89.2	67.2	52.2	.000
Mood	0.46**	**0.55****	0.22**	0.48**	−0.28**	0.27**	73.5	63.1	59.3	.017
Personality†	0.38**	**0.42****	0.15*	0.35**	−0.27**	0.14*	66.8	55.6	53.7	.043
Self–care	**0.62****	0.44**	0.25**	0.42**	−0.54**	0.21**	91.15	65.8	59.0	.000
Social roles	0.49**	0.45**	**0.30****	0.43**	−0.35**	0.19**	62.8	37.4	32.9	.000
Thinking†	0.38**	0.42**	0.13*	0.36**	−0.16**	**0.29****	66.2	57.4	63.5	.365
UE function	**0.57****	0.36**	0.27**	0.38**	−0.48**	0.24**	89.4	69.2	64.3	.000
Vision†	**0.33****	0.20**	0.16**	0.30**	**−0.19****	0.16**	97.3	89.9	88.9	.037
Work	**0.59****	0.42**	0.27**	0.36**	**−0.48****	0.18**	77.1	52.7	42.8	.000
Total score	**0.72****	**0.60****	**0.30****	**0.53****	−0.51**	0.33**	78.3	60.9	56.9	.000
**8–domain SSQoL**										
Energy	**0.49****	0.48**	0.15**	0.35**	−0.24**	0.28**	66.4	48.8	50.5	.007
Activities	**0.70****	0.46**	0.30**	0.44**	−0.58**	0.24**	87.2	63.1	53.3	.000
Language	0.29**	0.28**	0.21**	0.27**	**–0.29****	**0.27****	90.9	81.1	83.0	.011
Mood	0.47**	**0.56****	0.23**	0.45**	−0.27**	0.24**	70.7	63.0	53.6	.010
Social roles	0.50**	0.48**	**0.30****	0.45**	−0.36**	0.21**	66.1	41.6	38.4	.000
Total score	**0.72****	**0.59****	**0.32****	**0.53****	−0.52**	0.33**	79.8	62.3	57.9	.000

†:The same domain is included in the 8–domain SSQoL. Bold: the domains assumed to have correlations with HRQoL domains. BI: Barthel Index; IADL: instrumental activities of daily living; MMSE: Mini–Mental State Examination; NIHSS: National Institutes of Health Stroke Scale; SSQoL: Stroke–Specific Quality of Life Scale; WHOQoL–BREF: World Health Organization Quality of Life–BREF.

**P*<0.05; ***P*<0.01.

Downloaded from cre.sagepub.com at Fu Jen Catholic University, on October 29, 2014

Table 5. Convergent and discriminant validity of SIS 3.0 vs SIS–16.

Domains	**WHOQoL–BREF**				**NIHSS**	**MMSE**	**NIHSS**			
	Physical	Psychological	Social	Environment			Minor	Mild	Moderate	*P value*
	N=263						*N*=52	*N*=52	*N*=52	
**SIS 3.0**										
Strength	**0.54****	0.42**	0.31**	0.38**	−0.51**	0.16**	65.1	42.0	23.9	.000
Hand function	**0.46****	0.41**	0.26**	0.35**	−0.59**	0.14**	71.6	37.1	17.4	.000
Mobility	**0.61****	0.36**	0.23**	0.33**	−0.57**	0.24**	90.1	62.7	52.6	.000
ADL/IADL	**0.59****	0.45**	0.28**	0.41**	−0.57**	0.20**	86.2	64.1	54.3	.000
Memory and thinking	0.32**	0.29**	0.16**	0.36**	−0.17**	**0.32****	91.6	82.3	84.2	.019
Communication	0.24**	0.29**	0.19**	0.32**	**−0.26****	**0.29****	95.5	92.7	87.8	.032
Emotion	0.46**	**0.56****	0.20**	0.43**	−0.30**	0.30**	77.0	64.3	60.7	.000
Social participation	0.56**	0.47**	**0.27****	0.40**	−0.49**	0.22**	79.0	56.0	47.5	.000
Total score	**0.66****	**0.55****	**0.32****	**0.49****	−0.64**	0.29**	82.0	62.7	53.6	.000
Composite physical	**0.63****	0.47**	0.29**	0.41**	−0.64**	0.22**	81.8	55.7	42.8	.000
**SIS–16**	**0.62****	0.42**	0.25**	0.37**	−0.62**	0.24**	89.0	65.4	54.7	.000

Bold: the domains assumed to have correlations with HRQoL domains. BI: Barthel Index; IADL: instrumental activities of daily living; MMSE: Mini–Mental State Examination; NIHSS: National Institutes of Health Stroke Scale; SIS: Stroke Impact Scale; WHOQoL–BREF: World Health Organization Quality of Life–BREF. * $P<0.05$; **$P<0.01$.

Downloaded from cre.sagepub.com at Fu Jen Catholic University, on October 29, 2014

minor, mild, and moderate severity for the validation of discriminant validity. In summary, the four health-related quality of life measures have been compared with the sample characteristics well controlled.

The mean scores of the four health-related quality of life measures were slightly higher than the midpoint. All subscales of the four health-related quality of life measures had acceptable floor effects, except for the hand function domain of the Stroke Impact Scale 3.0. These four stroke-specific health-related quality of life measures showed prominent ceiling effects than floor effects. The ceiling effect can be explained by the fact that most patients did not show severe disease severity or severe levels of independence in terms of Barthel Index score. The 12-domain Stroke-Specific Quality of Life Scale or 8-domain Stroke-Specific Quality of Life Scale were slightly better than the Stroke Impact Scale 3.0 and Stroke Impact Scale-16 in meeting scaling assumptions, consistent with a previous study.[5] In summary, the ceiling effect was notable in the 4 health-related quality of life measures. These notable ceiling effects can possibly result from the recruitment of the patients not only with mild to moderate level of stroke but also those with minor severity. Thus, the items to reflect the health-related quality of life levels of the patients with minor, mild, to moderate severity of stroke are limited and are suggested to be expanded in future studies.

Repeated measurements require instruments to have reliability. The internal consistency of most of the domains of the four health-related quality of life measures was acceptable, as found in previous reports,[1,3,5,31] except for the domains of family roles and personality of the 12-domain Stroke-Specific Quality of Life Scale. In terms of test-retest reliability, no significant differences were detected between the health-related quality of life scores in the first and second administrations, except for the emotion domain of the Stroke Impact Scale 3.0. Only the social participation domain of the Stroke Impact Scale 3.0 had slightly low test-retest reliability. Overall, the four health-related quality of life measures are reliable, except for the family roles and personality domains of the 12-domain Stroke-Specific Quality of Life Scale and the social participation domain of the Stroke Impact Scale 3.0. From these perspectives, the 8-domain Stroke-Specific Quality of Life Scale show better reliability over the 12-domain Stroke-Specific Quality of Life Scale and Stroke Impact Scale 3.0 and thus is indicative of greater stability in regular use for clinical practitioners and researchers.

Acceptable precision i.e., absolute reliability was found in most of the domains of the four health-related quality of life measures. Only the standard error of measurements of the family role domain of the 12-domain Stroke-Specific Quality of Life Scale and the social participation domain of the Stroke Impact Scale 3.0 lacked acceptable precision. Similarly, all domains of the Stroke Impact Scale 3.0 met the criteria of the Standard error of measurement, except the emotion domain.[5] Moreover, the Standard error of measurement and Smallest real difference values of the composite physical domain of the Stroke Impact Scale 3.0 in this study are highly similar to those reported by Carod-Artal.[5] The smallest real difference values of ranged lowest value in the vision to the highest value in the family roles for the 12-domain Stroke-Specific Quality of Life Scale, from the lowest value in the activities to highest value in thinking for the 8-domain Stroke-Specific Quality of Life Scale, and from the lowest value in the ADL/Instrumental ADL activities to highest value in emotion for the Stroke Impact Scale 3.0. The smallest real difference value of the Stroke Impact Scale-16 was 13.2. Clinical change higher than these smallest real difference values alerts clinical practitioners and researchers to the possibility of true change in stroke survivors.

The construct validity was examined by investigating the convergent validity and discriminant validity. The four health-related quality of life subscales showed moderate to high correlations with the corresponding constructs from the World Health Organization Quality of Life-BREF Taiwan version, National Institutes of Health Stroke Scale, and Mini-Mental State Examination, except for the domains of family roles and vision of the 12-domain Stroke-Specific Quality of Life Scale. This study

Downloaded from cre.sagepub.com at Fu Jen Catholic University, on October 29, 2014

was the first to investigate the discriminant validity at 3 levels spanning minor to moderate severity, rather than only mild and moderate severity.[1] All four of the health-related quality of life subscales showed good discriminant validity, except for the thinking domains of the 12-domain Stroke-Specific Quality of Life Scale and 8-domain Stroke-Specific Quality of Life Scale. The discriminant validities of the 12-domain Stroke-Specific Quality of Life Scale and 8-domain Stroke-Specific Quality of Life Scale have never previously been confirmed for different levels of stroke severity. The memory and thinking domains of the Stroke Impact Scale 3.0 have been reported to have unsatisfactory discriminant validity in a report by Carod-Artal et al.[5] The Stroke Impact Scale-16 has shown good discriminant validity, as reported in the literature.[6,31,32] In summary, the four health-related quality of life measures showed sound convergent and discriminant validity. In other words, these four health-related quality of life measures can be suggested in the research and clinical use to reflect the subjective perspectives of disease impact on stroke patients' health-related quality of life levels and can be able to well differentiate different severity levels of stroke.

The number of stroke patients with severe severity recruited was limited. Similar limitation was also ever described by the report of Ewert et al.[3] Patients with severe stroke are less likely to be recruited due to the acute stage of disease or inability to complete self reports due to cognitive or communication impairment. The findings in the current studies have limited generalizability due to the recruitment in Taiwan, due to the stroke severity, low percentage of hemorrhagic type of stroke, and cognitive comprehension since patients were asked to complete the self-reports. Future studies can be improved by having the response rate, days after stroke, and side of lesion reported. Future studies also can have the patient characteristics compared between patients completed the retest and those who did not complete the retest. Increasing to compute the smallest real difference percentage (%) also can be helpful to the future comparisons of the 4 health-related quality of life measures. Future studies can compare these four measures with the other measures such as the newly developed Stroke-Specific Quality of Life Scale with 12 items.[33–35] Alternatively, future studies can have the versions of the Stroke-Specific Quality of Life Scale or the Stroke Impact Scale modified since refinement is indicated for family role domain of the 12-domain Stroke-Specific Quality of Life Scale, vision domain of the 12-domain Stroke-Specific Quality of Life Scale/8-domain Stroke-Specific Quality of Life Scale, and social participation domain of the Stroke Impact Scale 3.0. Also, reduction is indicated for the item numbers of high Cronbach's alpha values in the Stroke Impact Scale 3.0 and also indicated for the physical domains of the four health-related quality of life measures.

In summary, the four health-related quality of life measures showed acceptable psychometric properties, although their acceptability is less than satisfactory. Overall, as the hypothesis expected the use of 8-domain Stroke-Specific Quality of Life Scale and Stroke Impact Scale 3.0 are supported with sound psychometric properties and are feasible for clinical practice to monitor the disease-specific health-related quality of life levels of stroke survivors.

Clinical messages

- The 8-domain Stroke-Specific Quality of Life Scale is suggested as a good instrument choice for the clinical use to monitor the stroke-specific health-related quality of life levels.
- The Stroke Impact Scale 3.0 is suggested as a good instrument choice for the clinical use to monitor the stroke-specific health-related quality of life levels.

Acknowledgements

Appreciation is given to all authors for permission to use the measures, and to all raters and patients for participation.

Conflicts of interest

The authors declare no conflict of interest.

Downloaded from cre.sagepub.com at Fu Jen Catholic University, on October 29, 2014

Funding

This study was supported by research grants from Taiwan National Science Council (NSC 97-2314-B-030-005-MY2).

References

1. Williams LS, Weinberger M, Harris LE, Clark DO and Biller J. Development of a stroke-specific quality of life scale. *Stroke* 1999; 30:1362–1369.
2. Duncan PW, Wallace D, Lai SM, Johnson D, Embretson S and Laster LJ. The stroke impact scale version 2.0. Evaluation of reliability, validity, and sensitivity to change. *Stroke* 1999; 30: 2131–2140.
3. Ewert T and Stucki G. Validity of the SS-QOL in Germany and in survivors of hemorrhagic or ischemic stroke. *Neurorehabil Neural Repair* 2007; 21:161–168.
4. Duncan PW, Bode RK, Lai SM and Perera S. Rasch analysis of a new stroke-specific outcome scale: the Stroke Impact Scale. *Arch Phys Med Rehabil* 2003; 84: 950–963.
5. Carod-Artal FJ, Coral LF, Trizotto DS and Moreira CM. The stroke impact scale 3.0: evaluation of acceptability, reliability, and validity of the Brazilian version. *Stroke* 2008; 39: 2477–2484.
6. Duncan PW, Lai SM, Bode RK, Perera S and DeRosa J. Stroke Impact Scale-16: A brief assessment of physical function. *Neurology* 2003; 60: 291–296.
7. Hsueh IP, Jeng JS, Lee Y, Sheu CF and Hsieh CL. Construct Validity of the Stroke-Specific Quality of Life Questionnaire in Ischemic Stroke Patients. *Arch Phys Med Rehabil* 2011; 92: 1113–1118.
8. Lin KC, Fu T, Wu CY, Hsieh YW, Chen CL and Lee PC. Psychometric comparisons of the Stroke Impact Scale 3.0 and Stroke-Specific Quality of Life Scale. *Qual Life Res* 2010; 19: 435–443.
9. WHOQoL Group. Development of the World Health Organization WHOQoL-BREF quality of life assessment. *Psychol Med* 1998; 28: 551–558.
10. Yao G, Chung CW, Yu CF and Wang JD. Development and verification of validity and reliability of the WHOQOL-BREF Taiwan version. *J Formos Med Assoc* 2002; 101: 342–351.
11. Brott T, Adams HP, Olinger CP, Marler JR, Barsan WG, Biller J, et al. Measurements of acute cerebral infarction: a clinical examination scale. *Stroke* 1989; 20: 864–870.
12. Fischer U, Baumgartner A, Arnold M, Nedeltchev K, Gralla J, De Marchis GM, et al. What is a minor stroke? *Stroke* 2010; 41:661–666.
13. Chang KC and Tseng MC. Costs of acute care of first-ever ischemic stroke in Taiwan. *Stroke* 2003; 34: e219–e221.
14. Dewey HM, Donnan GA, Freeman EJ, Sharples CM, Macdonell RA, McNeil JJ, et al. Interrater reliability of the National Institutes of Health Stroke Scale: rating by neurologists and nurses in a community-based stroke incidence study. *Cerebrovasc Dis* 1999; 9: 323–327.
15. Goldstein LB and Samsa GP. Reliability of the National Institutes of Health Stroke Scale. Extension to non-neurologists in the context of a clinical trial. *Stroke* 1997; 28: 307–310.
16. Folstein MF, Folstein SE and McHugh PR. "Mini-mental state", a practical method for grading the cognitive state of patients for the clinician. *J Psychiatr Res* 1975; 12: 189–198.
17. Mahoney FI and Barthel DW. Functional Evaluation: The Barthel Index. *Md State Med J* 1965; 14: 61–65.
18. Carod-Artal FJ, Trizotto DS, Coral LF and Moreira CM. Determinants of quality of life in Brazilian stroke survivors. *J Neurol Sci* 2009; 15: 63–68.
19. Hsueh IP, Lee MM and Hsieh CL. Psychometric characteristics of the Barthel activities of daily living index in stroke patients. *J Formos Med Assoc* 2001; 100: 526–532.
20. Hsueh IP, Lin JH, Jeng JS and Hsieh CL. Comparison of the psychometric characteristics of the functional independence measure, 5 item Barthel index, and 10 item Barthel index in patients with stroke. *J Neurol Neurosurg Psychiatry* 2002; 73: 188–190.
21. van der Linden FA, Kragt JJ, Klein M, van der Ploeg HM, Polman CH and Uitdehaag BM. Psychometric evaluation of the multiple sclerosis impact scale (MSIS-29) for proxy use. *J Neurol Neurosurg Psychiatry* 2005; 76: 1677–1681.
22. Parent EC, Hill D, Moreau M, Mahood J, Raso J and Lou E. Score distribution of the Scoliosis Quality of Life Index questionnaire in different subgroups of patients with adolescent idiopathic scoliosis. *Spine* 2007; 32: 1767–1777.
23. Fayers PM and Machin D. Quality of life, the assessment, analysis and interpretation of patient-reported outcomes. 2nd edn. England: John Wiley & Sons Ltd, 2007.
24. Nunnally JC and Bernstein IH. *Psychometric Theory*, 3rd ed. New York: McGraw-Hill Inc, 1994.
25. Bland JM and Altman DG. Cronbach's alpha. *BMJ* 1997; 314: 572.
26. Lexell JE and Downham DY. How to assess the reliability of measurements in rehabilitation. *Am J Phys Med Rehabil* 2005; 84: 719–723.
27. Beckerman H, Roebroeck ME, Lankhorst GJ, Becher JG, Bezemer PD and Verbeek AL. Smallest real difference, a link between reproducibility and responsiveness. *Qual Life Res* 2001; 10: 571–578.
28. Wyrwich KW, Nienaber NA, Tierney WM and Wolinsky FD. Linking clinical relevance and statistical significance in evaluating intra-individual changes in health-related quality of life. *Med Care* 1999; 37: 469–478.
29. Portney LG and Watkins MP. *Foundations of Clinical Research: Applications to Practice*, 2nd edn. Upper Saddle River, NJ: Prentice-Hall Health, 2000.
30. Kerlinger FN. *Foundations of Behavioral Research*. New York: Holt, Rinehart and Winston, 1964.
31. Edwards B and O'Connell B. Internal consistency and validity of the Stroke Impact Scale 2.0 (SIS 2.0) and SIS-16 in an Australian sample. *Qual Life Res* 2003; 12: 1127–1135.

Downloaded from cre.sagepub.com at Fu Jen Catholic University, on October 29, 2014

32. Lai SM, Perera S, Duncan PW and Bode R. Physical and social functioning after stroke: comparison of the Stroke Impact Scale and Short Form-36. *Stroke* 2003; 34: 488–493.
33. Kerber KA, Brown DL, Skolarus LE, Morgenstern LB, Smith MA, Garcia NM, et al. Validation of the 12-item stroke-specific quality of life scale in a biethnic stroke population. *J Stroke Cerebrovasc Dis* 2013; 22: 1270–1272.
34. Post MW, Boosman H, van Zandvoort MM, Passier PE, Rinkel GJ and Visser-Meily JM. Development and validation of a short version of the Stroke Specific Quality of Life Scale. *J Neurol Neurosurg Psychiatry* 2011; 82: 283–286.
35. Wong GK, Lam SW, Ngai K, Wong A, Poon WS and Mok V. Development of a short form of Stroke-Specific Quality of Life Scale for patients after aneurysmal subarachnoid hemorrhage. *J Neurol Sci* 2013; 335: 204–209.

Downloaded from cre.sagepub.com at Fu Jen Catholic University, on October 29, 2014
